# Atypical Optic Neuritis as a Manifestation of Ocular Mucosa-Associated Lymphoid Tissue (MALT) Lymphoma: A Case Report

**DOI:** 10.7759/cureus.85856

**Published:** 2025-06-12

**Authors:** Carlos Sequeira Quesada, Maria Alexandra Vega Monge

**Affiliations:** 1 Neurology, Universidad de Costa Rica, San José, CRI; 2 Neurology, Caja Costarricense del Seguro Social (CCSS), San José, CRI; 3 Internal Medicine, Caja Costarricense del Seguro Social (CCSS), San José, CRI

**Keywords:** blindness, lymphoma, malt, neuritis, optic

## Abstract

Classic definitions describe optic neuritis as an acute, unilateral loss of vision accompanied by an afferent pupillary defect and eye pain, with normal ophthalmoscopy in most cases. Advancements in paraclinical tests have dichotomized clinical presentations into typical and atypical optic neuritis, with the latter requiring additional testing to establish a diagnosis of definite or probable optic neuritis. Compromise of the second cranial nerve in optic neuritis expands beyond inflammation and demyelination and includes systemic and infectious entities, more commonly catalyzing atypical clinical presentations. We present the case of a male patient with atypical optic neuritis, based on its clinical course and imaging findings, who was ultimately diagnosed with extranodal marginal zone B-cell lymphoma (EMZL), a type of non-Hodgkin MALT lymphoma. The effect of the optic nerve in this pathology has only been sparsely reported.

## Introduction

In 1991, the Optic Neuritis Study Group defined the typical profile of acute optic neuritis as unilateral vision loss, with afferent pupillary defect, and eye pain, with normal ophthalmoscopy in most cases [1].

Important advances and the development of new diagnostic resources have enhanced the ability to detect optic neuritis. Consensus in 2022 expanded the concepts involved in the definition of optic neuritis to include time of appearance (acute, subacute, or chronic), presence or lack of pain during extraocular movement, and monocular or binocular compromise. These clinical possibilities reflect the expanded differential diagnosis of optic nerve injury, with many new pathologies being discovered due to advancements in antibody testing and imaging modalities [1,2].

A dichotomy in clinical presentation of optic neuritis was instituted: typical and atypical optic neuritis. Variations from the classical description of optic neuritis are branded as red flags - or atypical optic neuritis [1,2].

To confirm a diagnosis of definite or possible optic neuritis, a combination of clinical criteria and paraclinical tests, such as Optical Coherence Tomography (OCT), Magnetic Resonance Imaging (MRI), and various biomarkers, is required. The closer the clinical presentation of the patient is to the classic definition established in 1991, the fewer additional abnormal paraclinical studies are needed to fulfill diagnostic criteria. Atypical neuritis on the counterpart needs more altered paraclinical tests, not only to establish a diagnosis of definite/possible optic neuritis but also to establish an inflammatory mechanism as the catalyzing factor, as over 60 different disorders can follow the diagnosis of optic neuritis, including systemic, infectious, and inflammatory entities [1,2].

We present the case of atypical optic neuritis that culminated in the rare diagnosis of mucosa-associated lymphoid tissue (MALT) lymphoma, involving not only the ocular adnexa but also extending to the optic nerve.

## Case presentation

The patient was a healthy 52-year-old male without any relevant family history or previous surgical interventions. He first sought medical consultation with Ophthalmology, presenting with non-painful, progressive monocular vision loss in the left eye, spanning back two years, and associated with conjunctival hyperemia. Isolated, severe monocular vision loss in the left eye and increased intraocular pressure were documented, and treatment for possible glaucoma was initiated.

Ancillary studies, such as OCT, were reported with diminished ganglion cells in the left eye, and the optic fundus showed increased excavation, with edema.

**Figure 1:**
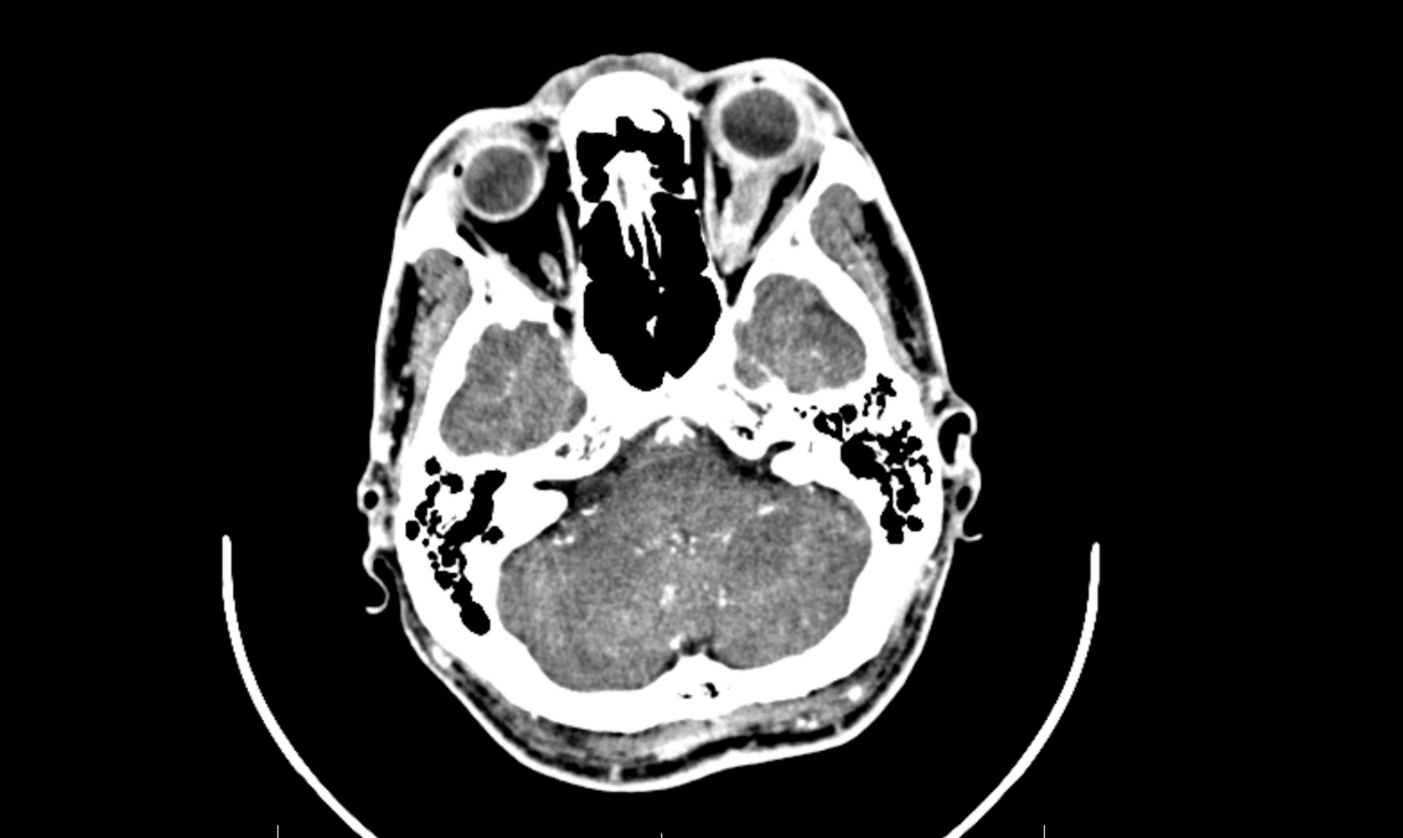
Axial contrast-enhanced computed tomography (CT) scan showing proptosis and an enlarged, enhancing optic nerve.

**Figure 2:**
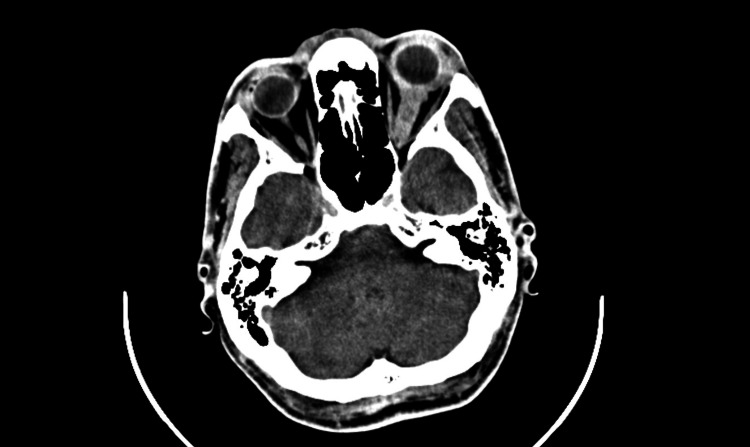
Axial non-contrast computed tomography (CT) scan showing proptosis and structural alterations of the left optic nerve.

Formal radiology report of non-contrast computer tomography (CT) indicated gorged and thickened arteries. Subsequent contrasted CT reported possible cellulitis and optic neuritis (Figures 1-2). Due to non-alignment between clinical evolution and imaging reports, consultation with neurology was solicited.

The Neurology team evaluated the patient with a working diagnosis of possible atypical optic neuritis, and further studies were conducted with the following results.

**Figure 3:**
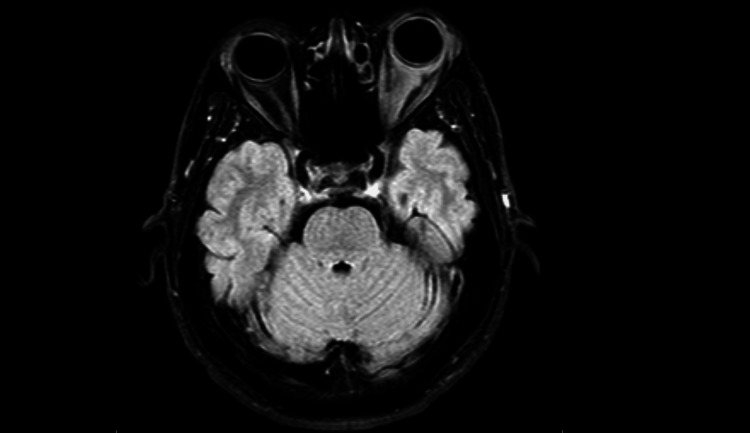
Axial fluid-attenuated inversion recovery (FLAIR) magnetic resonance imaging (MRI) showing structural changes in the left optic nerve, with no visible abnormalities in the brain parenchyma.

**Figure 4:**
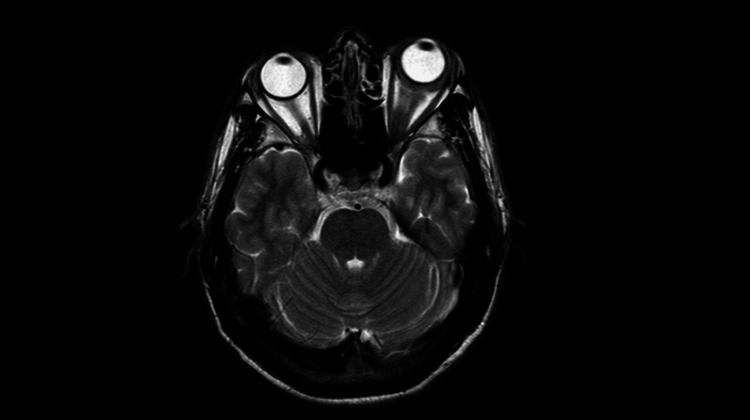
Axial T2-weighted magnetic resonance imaging (MRI) sequence showing structural changes in the left optic nerve, with no visible abnormalities in the brain parenchyma.

**Figure 5:**
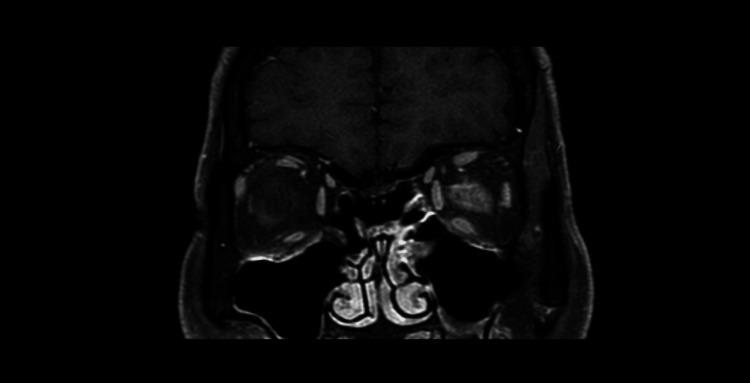
Coronal T2-weighted magnetic resonance imaging (MRI) showing structural changes in the left optic nerve, with no visible changes in the brain parenchyma.

MRI revealed significant thickening of the left optic nerve with gadolinium enhancement, also involving the adjacent periorbital fat and demonstrating diffusion restriction. Inflammatory involvement extended to the orbital apex (Figures 3-5).

HIV, cytomegalovirus, and Epstein-Barr virus tests were negative. VDRL was non-reactive, and all tested antibodies were negative, including antinuclear, antineutrophil cytoplasmic, anti-aquaporin-4, and anti-myelin oligodendrocyte glycoprotein antibodies. Lumbar puncture was normal, with no oligoclonal bands detected.

The patient received an empirical five-day course of intravenous methylprednisolone (1 g per day) without significant clinical improvement.

Abdominal ultrasound revealed no liver or spleen enlargement, and CT scan showed no lesions in the thorax, abdomen, or pelvis. Peripheral blood smear was normal, with no abnormalities in the hemogram.

**Figure 6:**
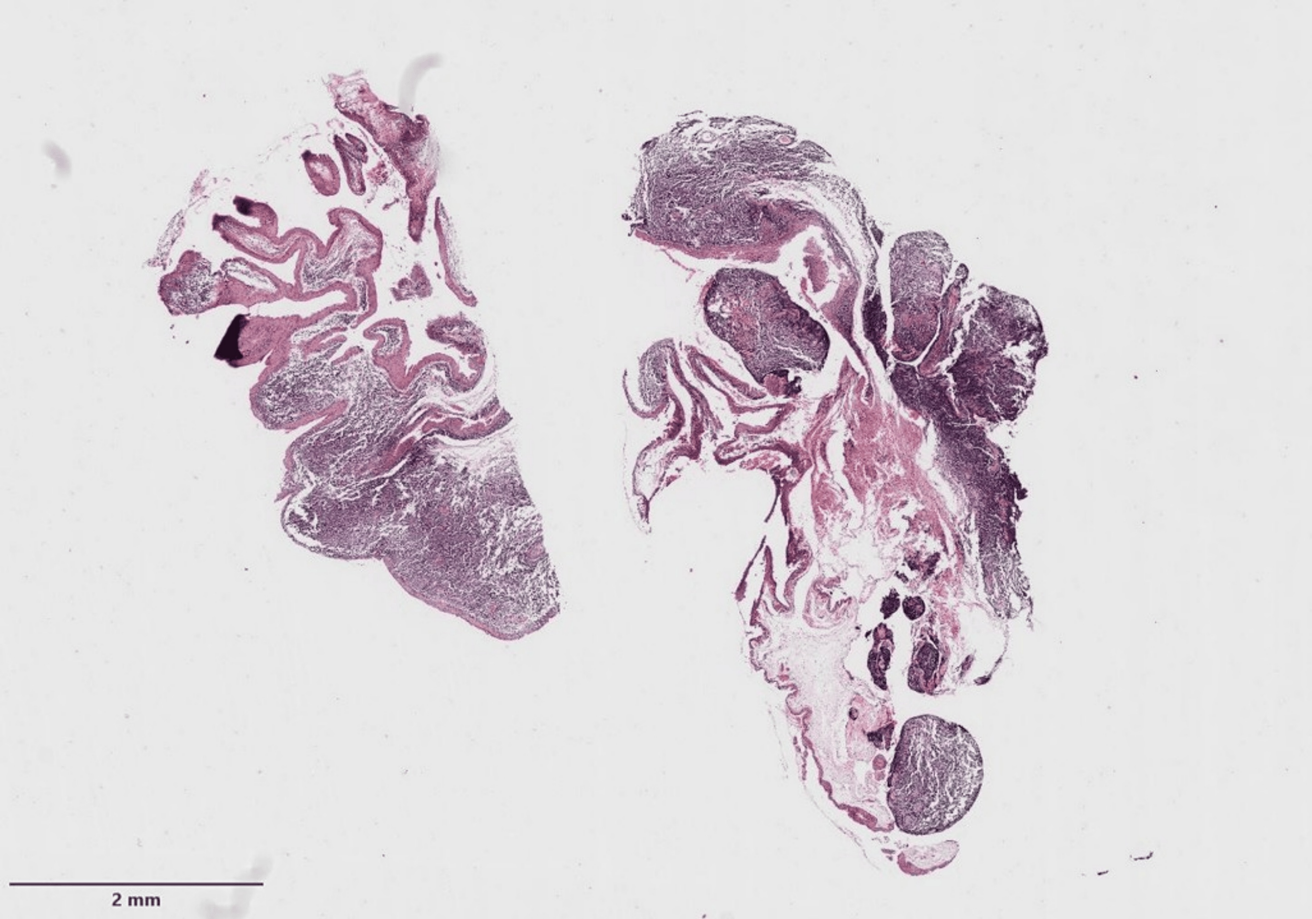
Histopathological analysis of the patient's conjunctival tissue showed expansion of the lamina propria due to a small-cell, monocytoid-like lymphoid cell population, with destruction of the normal architecture and remnants of the follicular dendritic cell network.

**Figure 7:**
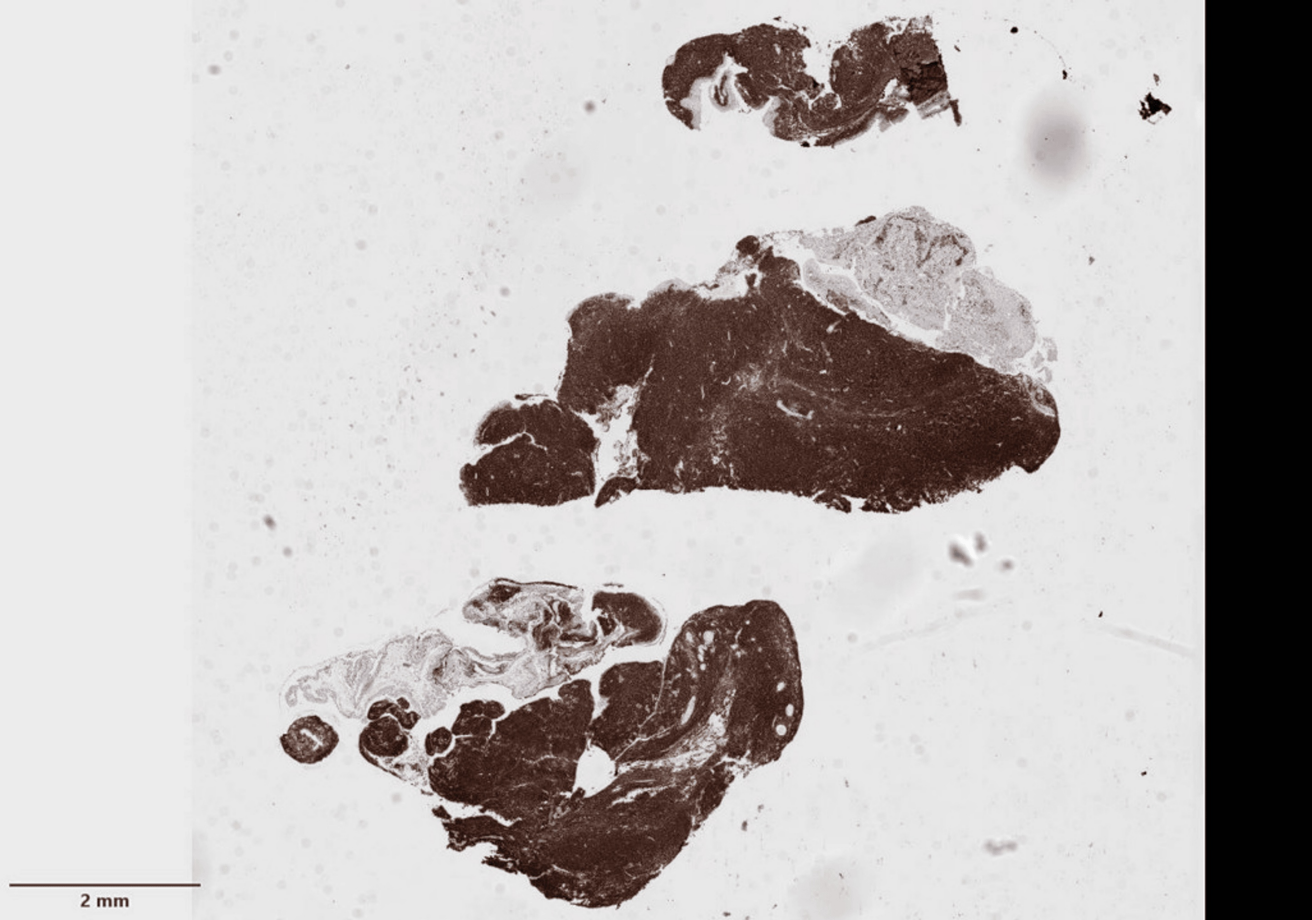
Histopathological sample of the patient's conjunctival tissue showing CD20-positive neoplastic cells.

**Figure 8:**
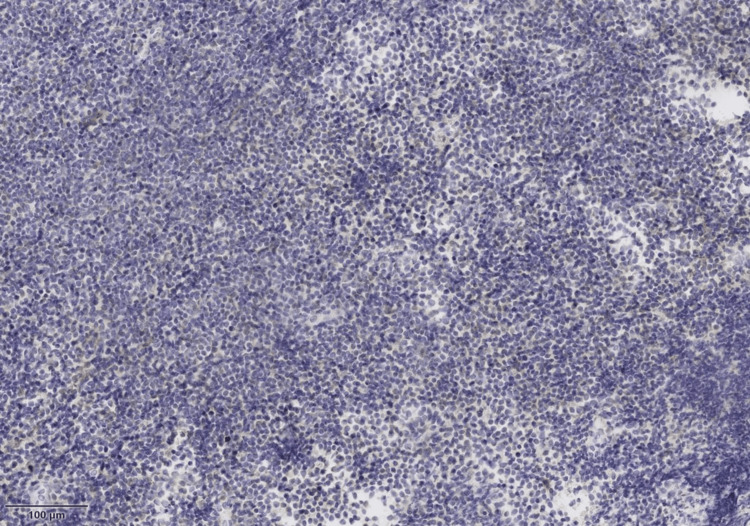
Histopathological sample of the patient's conjunctiva showed neoplastic cells negative for CD3, CD10, BCL6, CD5, CD23, cyclin D1, CD43, and MUM1.

The case was reevaluated with Ophthalmology, and a conjunctival biopsy was decided, with optic nerve biopsy deemed too risky. The histopathological sample was reported with lamina propria expansion due to a small-cell, monocytoid-like lymphoid cell population, with destruction of normal architecture and remnants of the dendritic follicular network. Neoplastic cells were CD20+, CD3-, CD10-, BCL-6-, BCL-2+, CD5-, CD23-, cyclin D1-, CD43-, and MUM-1-, with restriction of kappa chains and a 2% Ki-67 (Figures 6-8).

Hematology, therefore, confirmed the diagnosis of non-Hodgkin extranodal marginal zone B-cell lymphoma (EMZL), MALT type, staged as IAE according to the Ann Arbor Staging System, and initiated treatment with weekly rituximab and bendamustine every 21 days.

The patient completed four cycles of rituximab and three of bendamustine, without any major side effects during treatment and maintaining an Eastern Cooperative Oncology Group (ECOG) performance status of 0. The patient is currently awaiting follow-up neuroimaging to determine whether concomitant radiotherapy will be necessary.

## Discussion

Bibliographic review

Lymphomas are hematologic malignancies arising from the clonal expansion of B or T cells, and less commonly from natural killer (NK) cells. Lymphomas can occur in the conjunctiva, lacrimal gland, orbit, and eyelids. When they occur in the ocular adnexa, lymphomas can be classified as either primary tumors or secondary to systemic lymphoma involvement [3].

Among all extranodal presentations, ocular lymphomas account for approximately 5% of cases. [3] More than 95% are of B-cell origin, and 80% are low-grade. [4] The conjunctiva, as a barrier mucosa, contains specialized MALT, which functions to become activated in response to environmental antigens. The most common subtype of ocular lymphoma is EMZL, predominantly of the MALT type, occurring in 35%-80% of cases. It is followed by follicular lymphoma (20%), diffuse large B-cell lymphoma (DLBCL) (8%), and, less commonly, mantle cell lymphoma, small lymphocytic lymphoma, and lymphoplasmacytic lymphoma [5].

The predisposing factors for this condition have not been established. However, chronic inflammation due to persistent antigenic stimulation is thought to play a role, potentially leading to genetic abnormalities that result in the transformation of normal lymphoid cells into a MALT-type lymphoma. The nature of this chronic inflammatory process has not yet been identified, and autoimmunity as well as infections by Helicobacter pylori, Chlamydophila pneumoniae, and Chlamydophila psittaci have been proposed as causative agents [6].

The age of presentation is between the fifth and seventh decades of life, with a predominance in women. Eighty-five percent of patients are reported to be symptomatic at the time of diagnosis, with the main manifestations being increased ocular volume, irritation, and eyelid ptosis. Other less common manifestations include chemosis, hyperemia, dryness, epiphora, and photophobia. Most conjunctival lymphomas (90%) are macroscopically pink, characteristically described as salmon-shaped, typically mobile, and non-lobulated. In rare cases, they may originate primarily from uveal tissue, with nonspecific manifestations that can lead to initial misdiagnosis as uveitis, scleritis, or choroidal hemangioma [6]. The median time interval between symptom onset and diagnosis is variable, ranging from one month to 10 months.

It is extremely uncommon for this kind of tumor to spread to adjacent structures of the orbit. Therefore, compromise of visual acuity and visual fields has been described only sparsely in a few case reports and usually in association with rapidly growing tumors [7].

For diagnostic certainty, a biopsy should be performed with adequate tissue collection for anatomopathological and immunohistochemical analysis. The pathognomonic histological features include infiltration of germinal centers by malignant lymphocytes and the formation of lymphoepithelial lesions, resulting from the invasion of adjacent epithelial tissues by nests of MALT-type lymphoma cells. By immunolabeling, the neoplastic cells express B-cell antigens (CD20-CD79a), IgM, and in some cases IgG or IgA, with kappa or lambda light chain restriction, and are Bcl-2 positive. They can also express CD21 and CD35, markers that identify follicular dendritic cells. On the other hand, the neoplastic cells are negative for CD5, CD10, CD23, cyclin D1, and IgD [8,9].

Once the diagnosis is confirmed, extension studies should be conducted, including routine laboratory tests, protein electrophoresis, serum LDH, beta-2 microglobulin, CT scans of the chest, abdomen, and pelvis, and a bone marrow biopsy [4].

Therapeutic management is highly variable, and there are no standardized international guidelines that define a specific treatment approach. The choice of therapy depends primarily on two factors: the organ initially involved and the extent of the disease [10].

In some very localized cases, surgical resection may serve as therapy, but this management does not influence survival and increases the risk of recurrence. The watch-and-wait strategy has also been employed, primarily for older adults considered to be in fragile condition, particularly in cases of unilateral disease [11]. Radiotherapy is regarded as the gold standard treatment for isolated conjunctival lymphoma classified as Ann Arbor stage I [12].

In cases where conjunctival disease is accompanied by evidence of lymphoma at other sites, systemic chemotherapy with a combination of cyclophosphamide, hydroxydaunorubicin, vincristine, and prednisone (CHOP) is indicated. Rituximab has been used as first-line therapy for indolent ocular adnexal B-cell lymphoma [13]. In the largest randomized study of MALT lymphoma, the International Extranodal Lymphoma Study Group-19 (IELSG-19) study, a three-arm protocol compared six continuous weeks of chlorambucil, chlorambucil plus rituximab, and rituximab monotherapy. Better five-year survival was observed with the combined therapy (68% vs. 51%), but without a statistically significant difference in overall survival [14]. Regarding local therapy, there are a few reports of the use of Rituximab with partial response but fewer systemic adverse effects. Despite its association with Chlamydia psittaci infections, the use of doxycycline as first-line management is not recommended [15].

Extraintestinal MALT lymphomas have a very good prognosis, with overall survival rates of 90% at five years and 75%-80% at 10 years. Factors associated with poor prognosis include advanced age, impaired functional status, systemic symptoms, splenomegaly, and elevated levels of LDH or β2-microglobulin [10].

Clinical integration

The clinical course of the patient was defined as atypical due to the timing of presentation, absence of pain, overall progression, and conjunctival involvement. This classification was supported by abnormalities documented in imaging studies.

Ancillary studies revealed an alternative pathological mechanism unrelated to inflammation or demyelinating involvement, and a biopsy ultimately confirmed the diagnosis of EZML MALT lymphoma.

The patient did not present with any clinical signs or history suggestive of Chlamydia psittaci infection. Nonetheless, formal testing for this pathogen is not available at our center. As current evidence does not support antibiotic therapy as a first-line treatment [15], doxycycline was not offered.

Although this type of hematologic malignancy does not typically extend beyond the ocular adnexa or involve the optic nerve, previous case reports have documented this rare occurrence [16]. We present the first documented case of this clinical presentation in Costa Rica.

## Conclusions

Accurate clinical characterization of typical and atypical optic neuritis allows for appropriate expansion of ancillary studies to fulfill the criteria for definite optic neuritis and to identify the underlying cause. Uncommon presentations, such as EZML MALT lymphoma compromising the optic nerve, can be detected with this threshold.
